# 

**Published:** 1800-05

**Authors:** 


NEW MEDICAL PUBLICATIONS IN APRIL.

Medical Facts and Observations, volume the eighth, 4s. 6d. boards. Cadell.

Transactions of a Society for the improvement of Medical and Chirurgical Knowledge; vol. ii. 7s. 6d. boards. Johnson.

Considerations relative to Pulmonary Consumptions; by THOMAS SUTTON, M. D. Physician to the Forces, 3s. Robinsons.

Observations on the Effect of various Articles of the Materia Medica in the Cure of Lues Venerea; by JOHN PEARSON, senior Surgeon of the Lock Hospital and Asylum, and of the Public Dispensary, Reader on the Principles and Practice of Surgery, 4s. 6d. boards. Callow.

### NEW MEDICAL PUBLICATIONS IN GERMANY.

*Abhandlung über die Durchbohrung des Bruſtbeins:* i. e. A Treatiſe on Trepanning the Sternum: by C. F. Clossius. From the Latin. With a ſhort appendix, by I. G. Kramer, 8vo. pp. 72. *Marburg,* Academical Shop.

*Archiv für die Phyſiologie:* i. e. The Phyſiological Magazine: by Dr. I. C. Reil, Prof. in Halle, vol. III. 8vo. pp. 530, and four plates. *Halle,* Curt.

*Bibliothek für die Medicin, &c.* The Library; devoted to Medicine, Surgery, and Midwifery. By a Society of literary men; edited by F. Arneman, Prof. at Göttingen, 8vo. vol. I. *Göttingen,* Dietrich.

*Handbuch für die Mediciniſche Literatur, &c.* A Compendium of Medical Literature, in all its branches; or, an Introduction to the knowledge of the moſt valuable medical books, with their contents, prices, years of publication, and reviews; together with a variety of hiſtorical, biographical, and other remarks; ſyſtematically arranged. Attempted for the uſe of young phyſicians. By Dr. I. V. Rothe, 8vo. pp. 664. pr. 2 rixd. 6 gr. or about 9s. 6d. in ſheets. *Leipzig,* Kleefeld.

*Anleitung zum Auſstopfen, &c.* Inſtructions for filling and preſerving the coats of birds and mammillary animals. Derived from original principles and experimental rules, as well as thoſe of expert Naturaliſts. By G. Pistorius, (probably *Becker*) 8vo. 32 and 174 pp. Pr. 14 gr. or 2s. 6d. *Darmſtadt,* Heyer.

*Handbuch zur Kentniſs und Heilung, &c.* A Manual of the Theory and Practice of the internal Diſeaſes of the Human Body; compoſed chiefly in conſequence of original obſervations and experimental deductions made at the ſick-bed. By Dr. I. C. Stark, Prof. &c. &c. 8vo. pp. 46 and 668; with a well executed plate of the author. Pr. 2 rixd. 4 gr. or about 9s. *Jena,* Göpferdt.

---

### To CORRESPONDENTS.

*The unuſual influx of original communications this month (ſome of which we have been obliged to poſtpone, as well as the account of ſeveral valuable medical books which we have received) occaſions us to defer the articles from the foreign Journals till our next publication.*

*We acknowledge the receipt of communications from Drs. Vaughan and Carſon; Meſſrs. Peck, Shaw, Blackburn, Lipſcomb, Brown, and Moore.*

*We ſhall be much obliged to* R. H. *and* A Navy Surgeon, *for their ſignatures or addreſs.*

